# Effects of Balneotherapy in Jeju Magma-Seawater on Knee Osteoarthritis Model

**DOI:** 10.1038/s41598-020-62867-2

**Published:** 2020-04-20

**Authors:** Choong-Gon Kim, Dae-Geon Lee, Jina Oh, Youn-Ho Lee, Young Joon Lee, Phil Hyun Song, Chang-Hyun Song, Sae-Kwang Ku

**Affiliations:** 1 Marine Ecosystem and Biological Research Center, Korea Institute of Ocean Science and Technology, Busan, Republic of Korea; 2 Department of Anatomy and Histology, College of Korean Medicine, Daegu Haany University, Gyeongsan, Gyeongsangbuk-do Republic of Korea; 3 Department of Preventive Medicine, College of Korean Medicine, Daegu Haany University, Gyeongsan, Gyeongsangbuk-do Republic of Korea; 4 Department of Urology, College of Medicine, Yeungnam University, Daegu, Republic of Korea

**Keywords:** Preclinical research, Cartilage

## Abstract

Balneotherapy is a common non-pharmacological treatment for osteoarthritis (OA), however, the efficacy is controversial in knee OA. Jeju magma-seawater (JMS) has high contents of various minerals, which has anti-inflammatory and antioxidant properties via an oral route. Thus, we examined the effects of JMS bathing on knee OA and the combination effects with diclofenac sodium as an anti-inflammatory drug. Knee OA was induced by transection of the anterior cruciate ligament and the partial meniscectomy in rat. The rats were administered subcutaneously saline or diclofenac sodium in saline, followed by bathing in thermal distilled water or JMS for 8 weeks. The model represented the characteristic changes of the cartilage degradation, osteophyte formation and synovial inflammation, and the relevant symptoms of the joint swelling and stiffness. However, the JMS bathing reduced the joint thickness and improved the mobility. It also contributed to a well-preserved tissue supported by increases in bone mineral density of the joint and decreases in Mankin scores in the cartilages. The effects involved anti-inflammation, chondroprotection, anti-apoptosis, and chondrogenesis. Overall, the JMS bathing in combination with diclofenac sodium showed a similar trend associated with synergic effects. It suggests that JMS bathing can be promising for a clinical use in knee OA.

## Introduction

Osteoarthritis (OA), known as a degenerative joint disease, is one of the most common forms of arthritis^1^. In particular, knee joint is a load-bearing area primarily affected, and the knee OA accounts for more than 80% of the disease burden^2^. Knee OA is caused by a progressive degeneration of joint tissues including the cartilage, subchondral bone, synovial membrane and joint capsule^3^. The pathogenesis involves loss of the articular cartilage through death of chondrocyte and depletion of the cartilage components in the extracellular matrix (ECM), mainly proteoglycans and collagen type II, due to an imbalance between catabolic and anabolic activities^4^. Although the OA has been classified as a non-inflammatory arthritis, significant presence of synovitis in the patients suggests that inflammation is implicated in the pathogenesis^5^. Indeed, maladaptive pro-inflammatory pathways contribute to cell stress in the joint tissues and the ECM degradation^6^. The involvement of an inflammatory component is characterized by symptoms of joint pain, swelling and stiffness, and the following physical disabilities lead to a severely impaired quality of life. The prevalence of OA is related to aging, and several factors; systemic factors of dietary intake and bone density, and local factors of muscle weakness, obesity and joint laxity^1^. However, the exact causes involved in the multifactorial pathogenesis of knee OA are unclear, and the primary treatment is only partially effective and often associated with the adverse effects. Thus, there is a critical need to establish the therapeutic strategies for knee OA, along with increasing life expectancy and a rise of obesity.

Current management options for knee OA are based on non-pharmacological and pharmacological measures according to guidelines of the American College of Rheumatology and the OA Research Society International (OARSI)^7,8^. Non-pharmacological treatments include patient education, weight loss, moderate exercises, physical therapy and occupational therapy. Pharmacological recommendations include acetaminophen (paracetamol), non-steroidal anti-inflammatory drugs (NSAIDs), cyclooxygenase (COX)-2 inhibitors and opioid analgesics^7^. However, the clinical efficiency is limited to the symptomatic relief, and some pharmacological agents have unwanted adverse effects such as cardiovascular and gastrointestinal complications and renal failure^8–11^. Given that the risk of disease increases with age, careful consideration is required for treating of elderly patients with potential complication^12^. There have been recently urging interests in developing disease-modifying drugs to inhibit the OA progression with the symptomatic relief by targeting cartilage tissues, subchondral bone remodeling and inflammatory pathways^13^. Although the representative drugs, glucosamine and chondroitin sulfate, are not approved for treating of knee OA by regulatory bodies, the European Society for Clinical and Economic Aspects of Osteoporosis and Musculoskeletal Diseases advocates to use them for background maintenance treatment in the first step of the algorithm recommendation^14^.

Balneotherapy involves immersion in thermal natural mineral waters^15^, which is a common non-pharmacological approach for OA as a non-invasive and cost-effective measure^16^. Spa therapy which employs multiple treatments including balneotheray and hydrotherapy, often combined with massage, exercise, physical therapy or rehabilitation, is also prevalently used for treatment of knee OA^17,18^. The balneotherapy and spa therapy have shown clinical improvements on knee OA in the randomized controlled trials (RCTs)^19^ and systematic review and meta-analysis^20,21^. In addition, mud-bath therapy and Neydharting mud-pack provide beneficial effects on the quality of life, painful symptoms and functional capacities in patients with knee OA even in the follow-up studies lasting over time^22,23^. The OARSI guideline recommends balneotherapy and spa therapy for patients with multiple-joint OA and the relevant co-morbidities^8^. However, the effectiveness is uncertain in patients with knee OA only probably due to poor methodological quality, which needs additional large and well-designed RCTs. Several studies support the therapeutic mechanisms of balneotherapy for knee OA; thermal mineral waters containing cations of sodium (Na), potassium (K), calcium (Ca), and magnesium (Mg), and anions of sulfate, chlorine, and bicarbonate, inhibit inflammatory process and relieve the pain via modulation of lymphocyte proliferation and cytokine production^24–28^.

Jeju magma-seawater (JMS) is abundant underground seawater in Jeju Island, a volcanic island located in South Korea. It contains high contents of minerals, such as Na, Mg, Ca, K, zinc (Zn), manganese (Mn), strontium, selenium (Se), and vanadium (V), because of long-term intrusion of seawater through pored basaltic rocks^29^. The dietary intakes of JMS have been reported to have anti-inflammatory and antioxidant properties^30^, suggesting favorable effects of minerals contained in JMS. However, there have been no studies on effects of balneotherapy in JMS on knee OA to the best of our knowledge. Previously, we have shown that bathing in deep seawaters containing various minerals improve the atopic dermatitis^31,32^. *In vivo* and *in vitro* studies have established that water-soluble minerals can permeate a human skin^33,34^. Thus, we examined the bathing effects of JMS on knee OA, and its synergic effects in combination with diclofenac sodium as a COX-2 inhibitor.

## Results

### Changes on thickness of knee joint and joint capsule

**Figure 1:**
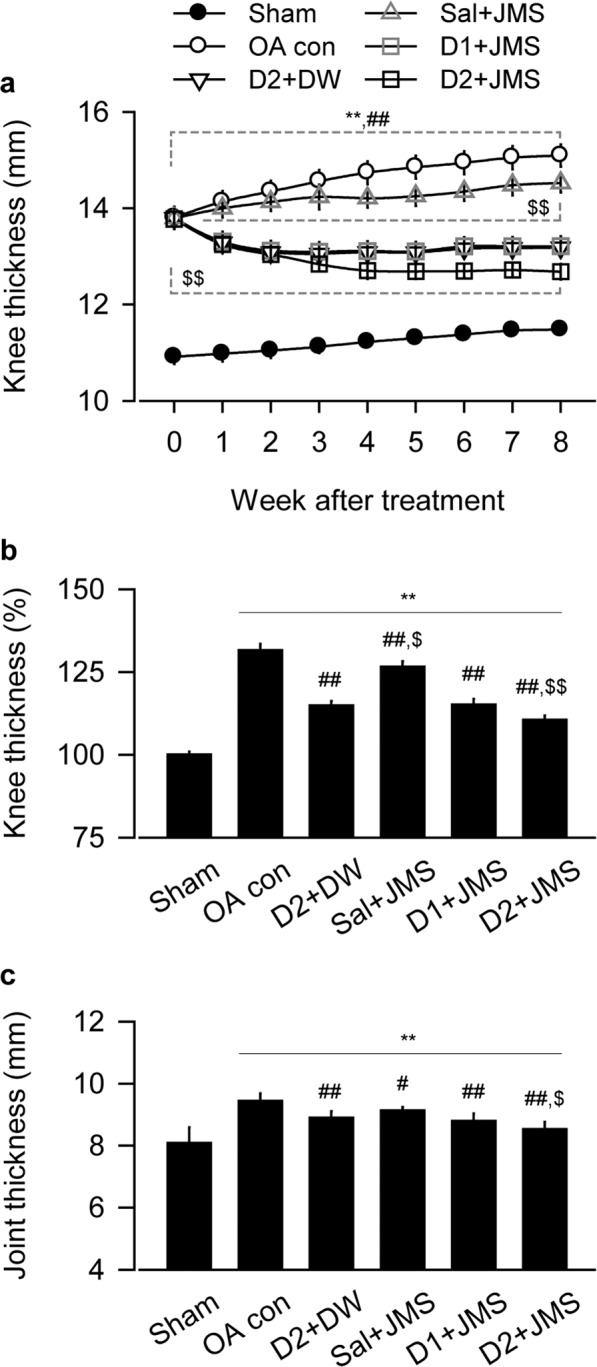
Thickness of knee joint and joint capsule. Knee osteoarthritis (OA) rat model received an injection of saline (Sal) or diclofenac sodium in saline at 1 mg/kg (D1) or 2 mg/kg (D2), followed by bathing in thermal distilled water (DW) or Jeju magma-seawater (JMS). The group was designated according to the treatment of injection plus bathing. The OA control and the corresponding sham received Sal plus DW bathing. The kinetic changes on knee thickness and the relatives to Sham group at 8 week after treatment are indicated in (**a**,**b**), respectively. After all treatments, thickness of joint capsule is shown in (**c**). Values were expressed as means ± standard deviation (SD: eight samples/group). The kinetic changes were examined by two-way ANOVA as repeated measurements, and the others were by one-way ANOVA. **p < 0.01 versus sham group, ^##^p < 0.01 and ^#^p < 0.05 versus OA con, and ^$$^p < 0.01 and ^$^p < 0.05 versus D2 + DW by LSD post-hoc tests.

Our knee OA model exhibited joint swelling and palpable synovitis, however, the inflammation-related symptoms seemed to be alleviated by the JMS bathing with diclofenac sodium at 1 mg/kg and 2 mg/kg in saline (D1 + JMS and D2 + JMS groups, respectively) compared with the knee OA negative control (OA con) treated with saline injection (Sal) plus bathing in distilled water (DW). The kinetic change of knee thickness for 8 weeks was examined by two-way analysis of variance (ANOVA). There were significant main effects for the groups (F = 346.2; p < 0.01) and the week measured (F = 130.3; p < 0.01). There were also significant interactions between the groups and week (F = 255.9; p < 0.01). The post-hoc tests versus the sham group showed significant increases in the thickness of all knee OA model regardless of the treatments (p < 0.05, Fig. 1a). However, compared with the OA con, the thickness was significantly decreased in the D2 + DW, D1 + JMS and D2 + JMS groups on weeks 1 to 8 post-treatment and in the Sal + JMS on weeks 2 to 8 (p < 0.05). In particular, the thickness was decreased more in the D2 + JMS group than the D2 + DW on weeks 3 to 8 (p < 0.05). Although it increased in knee OA model compared with sham group after treatments for 8 weeks, it significantly decreased in all treatment groups, the D2 + DW, Sal + JMS, D1 + JMS and D2 + JMS (p < 0.01, Fig. 1b). The thickness was measured directly in the joint capsule exposed from surrounding tissues after all treatments. One-way ANOVA showed significant main effects for the groups (F = 20.5; p < 0.01). The post-hoc test versus the sham group revealed increases in the capsule thickness of the OA con; however, the test versus the OA con showed significant decreases in all treatment groups (p < 0.05, Fig. 1c). Consistently, the capsule thickness was decreased more in the D2 + JMS group than the D2 + DW (p < 0.05). There were no body weight changes among groups (see Supplementary Fig. S1).

### Effects on the maximum extension angle of knee joint

**Figure 2:**
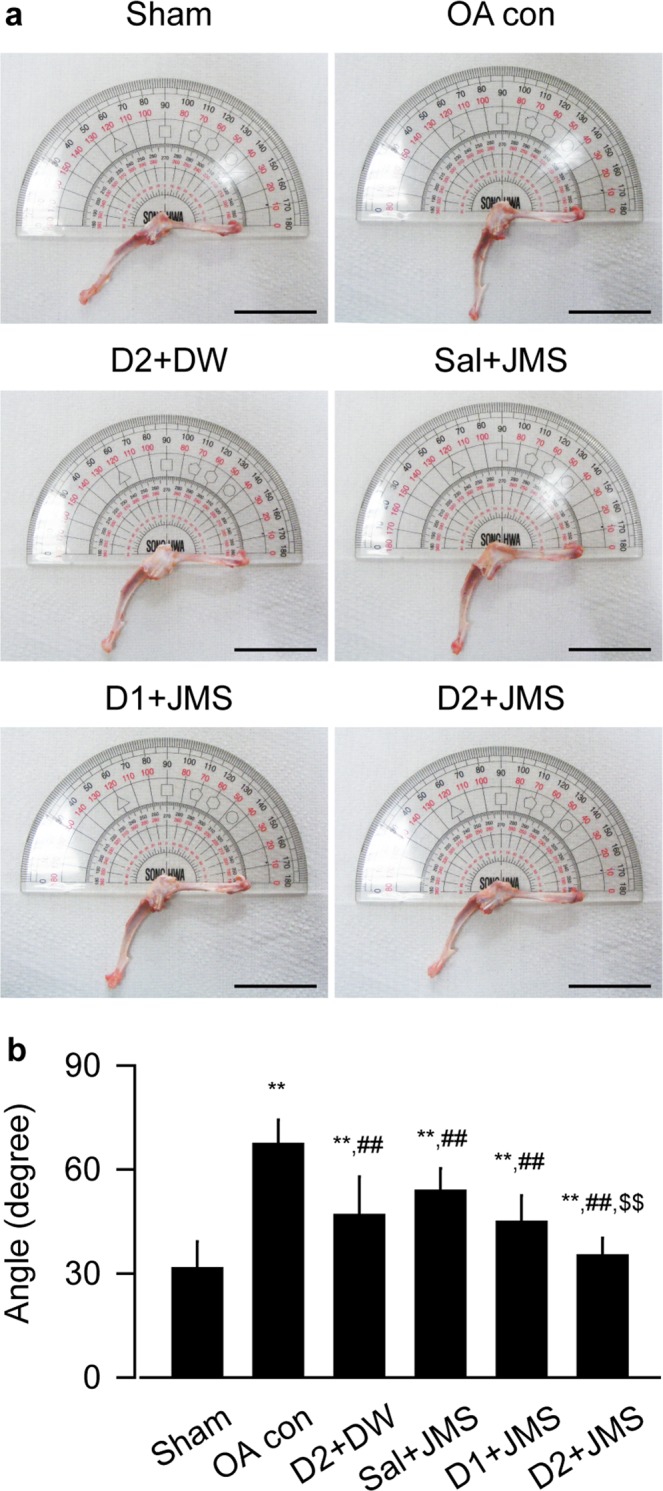
Maximum articular extension angle. Representative images for the maximum extension angle of knee joint were shown in (**a**). Scale bars = 36 mm. Values were expressed as means ± SD (eight samples/group) (**b**). **p < 0.01 versus sham group, ^##^p < 0.01 versus OA con, and ^$$^p < 0.01 versus D2 + DW by LSD.

The maximum extension angle was assessed for improvement of the knee joint stiffness. The angles appeared to be increased in the OA con group, indicating deteriorated knee mobility, but they seemed to be reduced in the treatment groups (Fig. 2a). One-way ANOVA showed significant main effects for the groups (F = 22.4; p < 0.01). The post-hoc test versus the sham group revealed significant increases in the maximum extension angles of the OA con; however, the test versus the OA con showed significant decreases in the treatment groups (p < 0.05, Fig. 2b). The angles were decreased more in the D2 + JMS group than the D2 + DW, suggesting the synergic effects of JMS bathing in combination with diclofenac sodium.

### Effects on bone mineral density (BMD) and compressive strength

**Figure 3:**
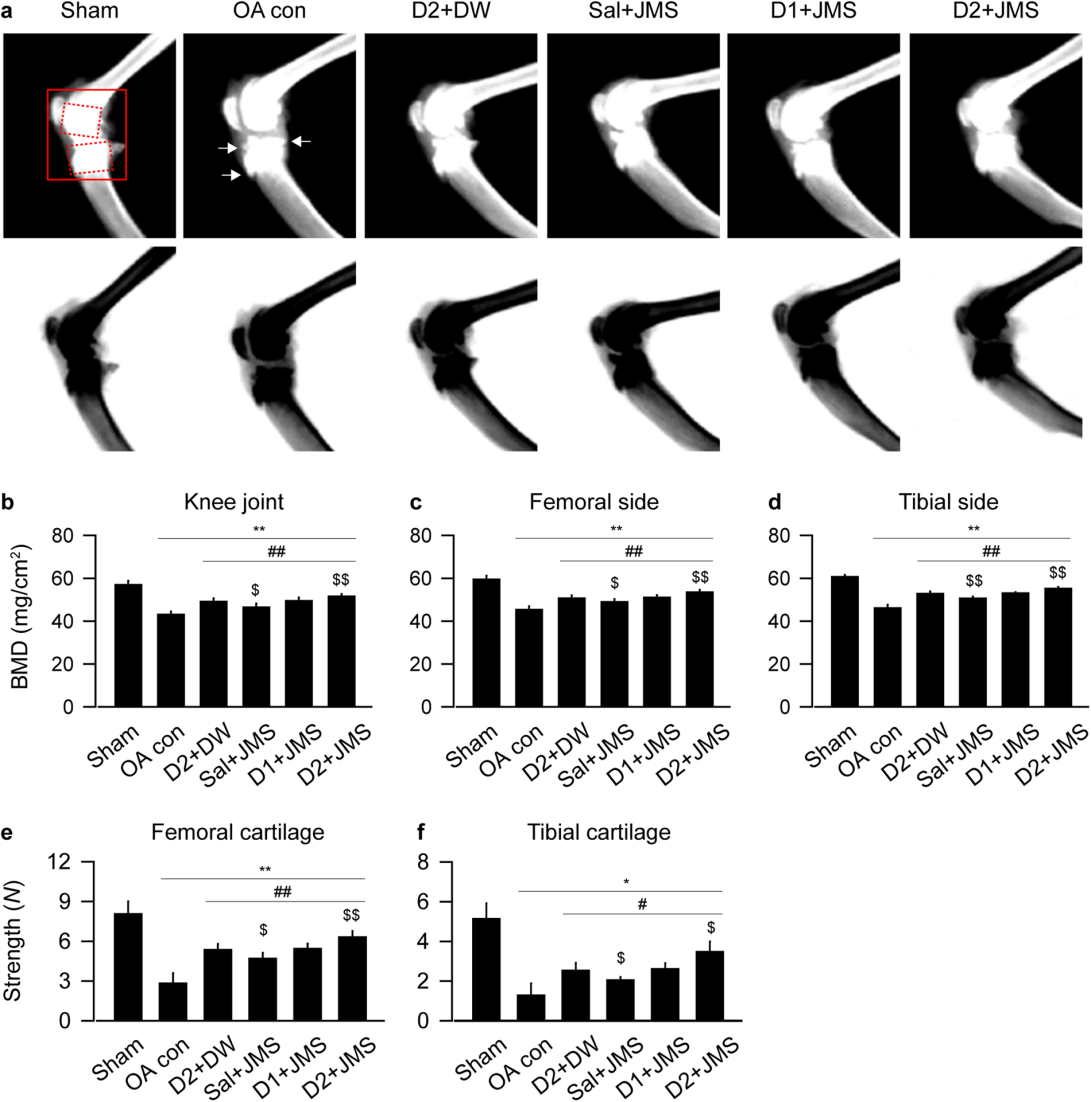
Bone mineral density (BMD) and compressive strength. BMD was assessed by dual energy X-ray absorptiometry digital radiography. The representative image and its reverse image are shown in (**a**) (upper and lower, respectively). Arrows indicate osteophytes. A region of interest is represented as solid and dotted boxes for the knee joint and both subchondral bones of the femur and tibia, respectively, and the BMDs were measured (**b**–**d**). Compressive strength was assessed in the cartilages of the femur (**e**) and tibia (**f**). Values were expressed as means ± SD (eight samples/group). **p < 0.01 and *p < 0.05 versus sham group, ^##^p < 0.01 and ^#^p < 0.05 versus OA con, and ^$$^p < 0.01 and ^$^p < 0.05 versus D2 + DW by LSD (**b**–**e**) or MW (**f**).

While dual-energy X-ray absorptiometry (DEXA) images of the knee joints depicted evident erosions and osteophyte formations in the OA con group, the lesions were mild in the treatment groups (Fig. 3a). For BMD of knee joint, one-way ANOVA showed significant main effects for group (F = 61.8; p < 0.01). The post-hoc test versus the sham group revealed decreases in the BMD of the OA con; however, the test versus the OA con showed significant increases in the treatment groups (p < 0.05, Fig. 3b). The data for BMD were further analyzed in both subchondral bones of the femur and tibia. One-way ANOVA showed significant main effects for the groups in both sides of the femur (F = 81.4; p < 0.01) and tibia (F = 147.0; p < 0.01). The post-hoc tests showed similar trends (Fig. 3c,d); lower in the OA con than the sham but higher in the treatment groups than the OA con (p < 0.05). Since BMD affects bone strength^35^, the compressive strength was assessed in both side cartilages (Fig. 3e,f). There were significant main effects for the groups in the femur (F = 63.4; p < 0.01, by ANOVA) and tibia (p < 0.01 by Kruskal-Wallis H). The post-hoc test versus the sham group revealed decreases in the compressive strength of both cartilages of the OA con, while the test versus the OA con showed significant increases in the treatment groups (p < 0.05). In particular, the data for BMD and compressive strength were higher in both cartilages of the D2 + JMS group than those of the D2 + DW (p < 0.05).

### Effects on inflammatory and proteolytic activities

**Figure 4:**
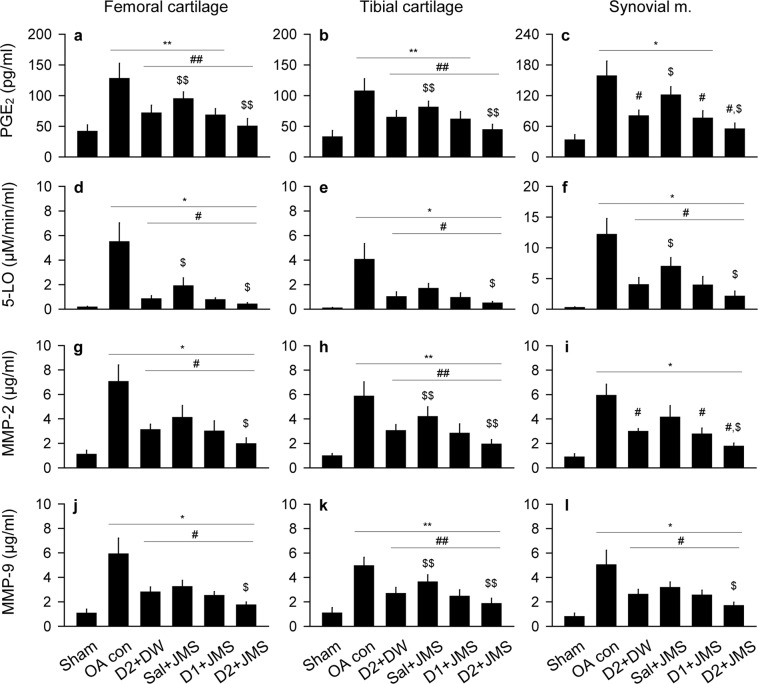
Expressions of inflammatory mediators and metalloproteinases (MMPs). Tissue levels of inflammatory cytokines, prostaglandin E_2_ (PGE_2_, **a**–**c**) and 5-lipoxygenase (5-LO, **d**–**f**), and MMPs, MMP-2 (**g**–**i**) and MMP-9 (**j**–**l**), were assessed in cartilages of the femur and tibia, and the synovial membrane (**m.**). Values were expressed as means ± SD (eight samples/group). **p < 0.01 and *p < 0.05 versus sham group, ^##^p < 0.01 and ^#^p < 0.05 versus OA con, and ^$$^p < 0.01 and ^$^p < 0.05 versus D2 + DW by LSD (**a**,**b**,**h**,**k**) or MW (**c**–**g**,**i**,**j**,**l**).

Levels of inflammatory mediators, prostaglandin E_2_ (PGE_2_) and 5-lipoxygenase (5-LO), and proteolytic enzymes, metalloproteinase (MMP)-2 and MMP-9, were assessed in both cartilages of the femur and tibia, and the synovial membrane (Fig. 4). The levels of PGE_2_ in both cartilages and the levels of MMP-2 and MMP-9 in the tibial cartilages were examined by one-way ANOVA and the others were by non-parametric Kruskal-Wallis H. The multiple comparison tests showed significant main effects for the groups (p < 0.01). For the levels of PGE_2_ and 5-LO, the post-hoc test versus the sham group revealed increases in both cartilages and the synovial membrane of the OA con (p < 0.05, Fig. 4a–f); however, the test versus the OA con showed significant decreases in the treatment groups (p < 0.05), excepting for PGE_2_ in the synovial membrane of the Sal + JMS group. The levels were significantly decreased more in the D2 + JMS group than the D2 + DW (p < 0.05). Similarly, while the levels of MMP-2 and MMP-9 in the cartilages and the synovial membrane were higher in the OA con group than the sham group, they were lower in the treatment groups than the OA con (p < 0.05), excepting for MMP-2 in the synovial membrane of the Sal + JMS group (Fig. 4g–l). The levels were further lower in the D2 + JMS group than the D2 + DW (p < 0.05).

### Effects on expressions of mRNA related to cartilage composition

**Figure 5:**
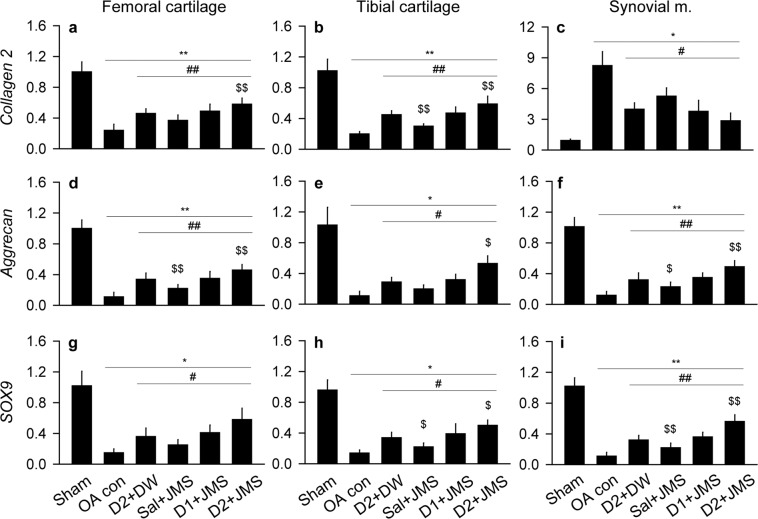
Expressions of mRNA related to cartilage composition. Relative mRNA expressions to β-actin were assessed in the cartilages and the synovial membrane. The expressions are for *collagen type II* (*collagen2*; **a**–**c**), *aggrecan* (**d**–**f**) and *sex-determining region Y-box (SOX)9* (g-i). Values were expressed as means ± SD (eight samples/group). **p < 0.01 and *p < 0.05 versus sham group, ^##^p < 0.01 and ^#^p < 0.05 versus OA con, and ^$$^p < 0.01 and ^$^p < 0.05 versus D2 + DW by LSD (**a**,**b**,**d**,**f**,**i**) or MW (**c**,**e**,**g**,**h**).

Expression levels of mRNA for collagen type II (collagen2) and aggrecan as key components for the cartilage ECM, and sex-determining region Y-box (SOX)9 as a transcription factor for chondrogenic differentiation, were assessed in both side cartilages and the synovial membrane (Fig. 5). One-way ANOVA examined data for the levels of *collagen2* in both cartilages, *aggrecan* in the femoral cartilage and synovial membrane, and *SOX9* in the synovial membrane, while non-parametric analyses examined for the others. There were significant main effects for the groups (p < 0.01), followed by the post-hoc tests. In both cartilages, the expressions of *collagen2*, *aggrecan* and *SOX9* were down-regulated in the OA con compared with the sham group; however, they were significantly up-regulated in the treatment groups compared with the OA con group (p < 0.05). In the synovial membrane, while the expression of *collagen2* was significantly higher in the OA con than the sham group, the other expressions of *aggrecan* and *SOX9* were lower (p < 0.05). However, the expressions were reversed in the treatment groups compared with the OA con group (p < 0.05). Furthermore, the levels of *collagen2*, *aggrecan* and *SOX9* were significantly higher in the cartilages of the D2 + JMS group than the D2 + DW, excepting for *SOX9* in the femoral cartilage. The levels of *aggrecan* and *SOX9* were also higher in the synovial membrane of the D2 + JMS than that of the D2 + DW (p < 0.05).

### Histopathological changes on cartilage degradation

**Figure 6:**
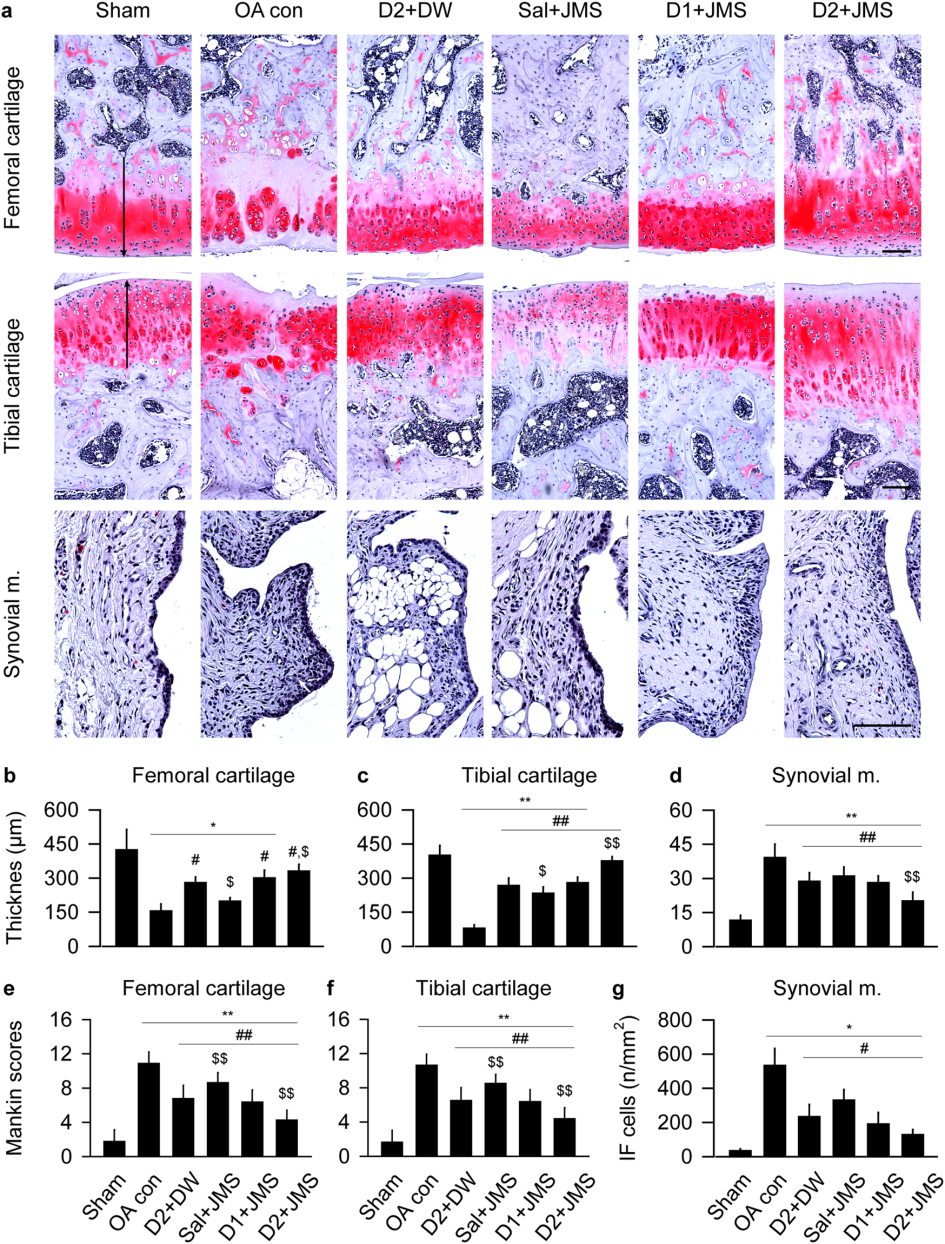
Histopathological analysis in Safranin O stain. Representative images are shown in the cartilages and the synovial membrane (m.) (**a**). Arrows indicate cartilage thickness of the femur and tibia. Scale bars = 120 µm. Thickness of cartilages of the femur (**b**) and tibia (**c**), and the synovial m. (**d**) were measured. In addition, total Mankin scores were assessed in both cartilages of the femur (**e**) and tibia (**f**). The maximum total score is 12. Inflammatory (IF) cells were counted in the synovial m. (**g**). Values were expressed as means ± SD (eight samples/group). **p < 0.01 and *p < 0.05 versus sham group, ^##^p < 0.01 and ^#^p < 0.05 versus OA con, and ^$$^p < 0.01 and ^$^p < 0.05 versus D2 + DW by LSD (**c**–**f**) or MW (**b**,**g**).

In Safranin O stain, the OA con group exhibited evident damages in a surface of the cartilages with the decreased chondrocyte, clone formation and stain intensity, however, the changes were mild in all treatment groups (Fig. 6a). The thickness of the tibial cartilage and synovial membrane and the Mankin scores in both side cartilages were examined by one-way ANOVA and the others were by Kruskal-Wallis H. The multiple comparison tests showed main effects for the groups (p < 0.01). The post-hoc versus the sham group revealed significant decreases in the thickness of both cartilages of the OA con; however, the test versus the OA con showed significant increases in the treatment groups, excepting for the femoral cartilages of the Sal + JMS (Fig. 6b,c). While total Mankin scores of both cartilages were higher in the OA con than the sham group, they were lower in the treatment groups than the OA con (p < 0.05, Fig. 6e,f). Conversely, the synovial membrane-lining epithelial thickness and inflammatory cells were increased in the OA con compared with the sham group; however, they were decreased in the treatment groups compared with the OA con (p < 0.05, Fig. 6d,g). In particular, the D2 + JMS group versus D2 + DW showed significant increases in the thickness of both cartilages and decreases in the Mankin scores and the thickness of the synovial membrane (p < 0.05).

### Immunohistochemical analyses on inflammation, apoptosis and cellular proliferation

**Figure 7:**
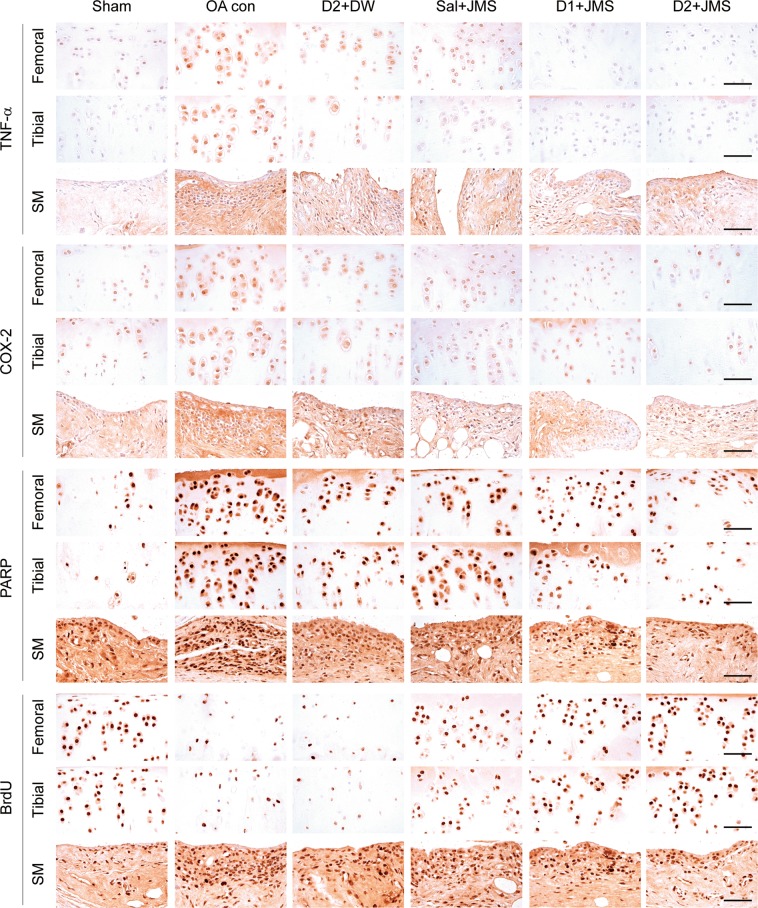
Immunohistochemistry for inflammation, apoptosis and cell proliferation. Another serial-section was immunostained for tumor necrosis factor (TNF)-α, cyclooxygenase (COX)-2, poly (ADP-ribose) polymerase (PARP) and bromodeoxyuridine (BrdU). The representative images were shown in the femoral and tibial cartilages (Femoral and Tibial, respectively), and synovial membrane (SM). Scale bars = 120 µm.

In immunostains for tumor necrosis factor (TNF)-α and COX-2 as pro-inflammatory markers and cleaved poly (ADP-ribose) polymerase (PARP) as an apoptosis marker, the expressions were less in all treatment groups than the OA con (Fig. 7). The immunoreactivities for bromodeoxyuridine (BrdU) as a cellular proliferation marker, were different in the cartilages and synovial membrane of the treatment groups compared with the OA con; increased in the cartilages but reduced in the synovial membrane. The COX2- and BrdU-positive cells in the femoral cartilage and tibial cartilage, respectively, were examined by one-way ANOVA, and the others were by Kruskal-Wallis H (Table 1). There were significant main effects for the groups (p < 0.01). The post-hoc versus the sham group revealed increases in the immunoreactive cells for TNF-α, COX-2 and PARP in both cartilages and the synovial membranes of the OA con; however, the test versus the OA con showed significant decreases in those of the treatment groups (p < 0.05), excepting for the tibial cartilage of the Sal + JMS group. Comparing to the D2 + DW group, the D2 + JMS group showed significant decreases in the immunoreactive cells in both cartilages and the TNF-α- and PARP-positive cells in the synovial membrane (p < 0.05). For the BrdU-positive cells, the post-hoc versus the sham group showed decreases in both cartilages of the OA con but increases in the synovial membrane (p < 0.05). However, the immunoreactivities were reversed in the Sal + JMS, D1 + JMS and D2 + JMS groups compared with the OA con (p < 0.05). The BrdU-positive cells were not different in both cartilages between the D2 + DW and the OA con groups; however, they were reduced in the synovial membrane of the D2 + DW compared with that of the OA con (p < 0.05).

**Table 1:** Immunohistochemical analysis for inflammation, apoptosis and cellular proliferation.

	Sham	OA con	D2 + DW	Sal + JMS	D1 + JMS	D2 + JMS
***TNF-α-positive cells***						
Femoral cart.	57.9 ± 15.1	336.4 ± 82.8^*^	135.9 ± 21.5^*,#^	238.9 ± 25.0^*,#,$^	126.9 ± 22.1^*,#^	97.4 ± 16.4^*,#,$^
Tibial cart.	44.8 ± 14.0	317.0 ± 58.2^*^	167.0 ± 29.8^*,#^	222.5 ± 44.6^*^	145.8 ± 20.2^*,#^	107.1 ± 15.6^*,#,$^
Synovial m.	16.3 ± 3.1	630.1 ± 132.4^*^	213.1 ± 44.1^*,#^	351.4 ± 100.5^*,#^	191.3 ± 37.0^*,#^	118.8 ± 22.6^*,#,$^
***COX2-postive cells***						
Femoral cart.	66.1 ± 22.3	405.4 ± 27.7^**^	162.8 ± 25.8^**,##^	231.8 ± 51.1^**,##,$$^	150.1 ± 37.3^**,##^	104.4 ± 14.6^**,##,$$^
Tibial cart.	92.3 ± 22.6	355.3 ± 106.0^*^	180.3 ± 27.8^*,#^	233.4 ± 34.4^*^	170.9 ± 31.6^*,#^	128.0 ± 26.2^#,$^
Synovial m.	27.5 ± 10.2	473.6 ± 82.8^*^	166.0 ± 29.1^*,#^	247.5 ± 32.4^*,#,$^	144.8 ± 22.7^*,#^	116.0 ± 31.6^*,#^
***PARP-postive cells***						
Femoral cart.	73.1 ± 15.2	470.0 ± 119.6^*^	171.0 ± 26.9^*,#^	323.4 ± 60.3^*,#,$^	164.5 ± 25.1^*,#^	122.3 ± 22.6^*,#,$^
Tibial cart.	56.6 ± 17.0	396.8 ± 107.5^*^	193.0 ± 46.2^*,#^	259.6 ± 25.8^*^	172.6 ± 36.1^*,#^	125.1 ± 24.6^*,#,$^
Synovial m.	79.6 ± 23.7	885.8 ± 153.1^*^	224.8 ± 40.1^*,#^	389.6 ± 66.7^*,#^	212.0 ± 57.6^*,#^	117.5 ± 15.7^*,#,$^
***BrdU-positive cells***						
Femoral cart.	128.0 ± 32.6	41.1 ± 12.09^*^	42.9 ± 10.4^*^	72.9 ± 11.0^*,#,$^	96.3 ± 15.6^#,$^	143.1 ± 12.9^#,$^
Tibial cart.	95.5 ± 29.8	31.3 ± 11.6^**^	32.9 ± 11.6^**^	62.6 ± 14.5^**,##,$$^	83.5 ± 12.7^##,$$^	115.3 ± 19.8^*,##,$$^
Synovial m.	84.1 ± 25.6	916.1 ± 178.2^*^	348.9 ± 147. ^*,#^	596.8 ± 119.9^*,#^	301.0 ± 73.8^*,#^	173.5 ± 26.7^*,#,$^

The immunoreactive cells in Fig. 7 were counted and expressed as means ± standard deviation (eight samples/group) in the femoral and tibial cartilages (cart.) and the synovial membrane (m.). **p < 0.01 and *p < 0.05 versus sham group, ^##^p < 0.01 and ^#^p < 0.05 versus OA con, and ^$$^p < 0.01 and ^$^p < 0.05 versus D2 + DW by LSD (for positive cells for COX2 of the femoral cartilages and BrdU of the tibial cart.) or MW (for others). TNF-α = tumor necrosis factor, COX2 = cyclooxygenase 2, PARP = poly (ADP-ribose) polymerase, and BrdU = bromodeoxyuridine.

## Discussion

Our knee OA model exhibited the characteristic changes of articular cartilage degradation, subchondral bone remodeling, osteophyte formation and synovial inflammation, and the relevant symptoms of joint swelling and stiffness. However, the JMS bathing alone (Sal + JMS group) inhibited the OA progress − reduced thickness of the knee and joint; improved articular motion; increased BMD in the joint and compressive strength of the cartilages. The histopathological analyses revealed reduced Mankin scores with increased thickness in the articular cartilages, and alleviated thickening of the synovial membrane. The favorable effects involved reduction of proinflammatory cytokines (PGE_2_, 5-LO, TNF-α and COX-2) and the cartilage ECM-degrading enzymes (MMP-2 and MMP-9). In addition, while the apoptotic (PARP-positive) cells were reduced in the articular tissues, the proliferative (BrdU-positive) cells were increased with up-regulation of chondrogenic genes (*SOX9, collagen2* and *aggrecan*). The overall effects of JMS bathing were significantly more so when combined with diclofenac sodium. It suggests the therapeutic potential of JMS bathing for knee OA.

The characteristic changes of knee OA lesions were observed in the DEXA and histopathological analyses, which were mild in treatments including the JMS bathing. An inflammatory mechanism has been noted as an important factor for promoting the structural damages in OA joints, as well as pain and immobility^36^. The initial change is characterized by infiltration of leukocytes in the osteoarthritic synovium^5,36^. The progress increases COX-2 and 5-LO in coordination with released cytokines including interleukin-1 and TNF-α, followed by production of PGE_2_ and leukotriene. It can cause pains and subchondral sclerosis progressively over time. Here, the JMS bathing inhibited the release of inflammatory mediators in the knee joint, which might contribute to alleviating the joint swelling and increasing the range of motion. The anatomical and physiological changes could be triggered by metabolic and molecular alterations in the joint. Along with cytokines, MMPs are also responsible for degeneration of joint cartilage through the ECM degradation^4^. On the other hands, SOX9 is a master regulator of chondrogenesis, and it regulates other chondrogenic genes, *collagen2* and *aggrecan*^37^. Particularly, MMP-2 and -9 degrade collagen denatured by collagenases and cleave aggrecan molecules^38^. In this context, the facts that JMS bathing reduced the MMPs and up-regulated the chondrogenic genes, might inhibit the physiological remodeling and pathological degeneration of the cartilage tissue. These provide useful information for protective effects of the JMS bathing on the pathological progress of knee OA.

The protective effects of the JMS bathing may contribute to a well-preserved cartilage tissue serving as a scaffold for mineral deposition and enhancing the proliferating and antiapoptotic activities. The increased subchondral BMD and chondrogenesis have positive correlations with the mechanical loading^35^. Further, the chondrogenic genes, collagen2 and aggrecan, can provide bony tensile and the compressive strength, respectively, as well as chondrocyte differentiation^39^. NSAIDs including diclofenac sodium produce pain relief and improvement of joint motion in knee OA, but some can be detrimental to bone regeneration by suppressing synthesis of proteoglycans^40^. Here, the proliferating cells in the cartilage were not changed by the treatment of diclofenac sodium followed by DW bathing. However, the proliferation was further increased by a combination with the JMS bathing. In other results as well, the combination treatments showed a similar trend associated with synergic effects. This suggests that JMS bathing in combination with a proper dose of diclofenac sodium can be promising as an ideal regimen for knee OA.

Some clinical trials provide compelling evidences for significant mineral effects on knee OA in balneotherapy in comparison with the pretreatment or tap water^41,42^. In addition, balneotherapy containing various minerals including bicarbonate, calcium and sulfur, involves reducing serum inflammatory cytokines (i.e., IL-1β, TNF-α, IL-6, IL-8, TGF-β), exerting inhibition of the cartilage degradation and pain relief^24–28,43^. The direct mineral effect on OA progression has been examined via the dietary intakes. Supplements including Mg, Mn, V and Se, alleviate the OA lesions and the relevant symptoms in the human patients and animal models^44–46^. In particular, Mg has received attention because it promotes chondrocyte viabilities and its deficiency involves outbreaks of knee OA or inflammatory responses^47,48^. Other animal mechanism studies have shown that dietary deep seawater containing NaCl, Mg, and Ca increases BMD and stimulates bone formation and resorption for a bone turnover^49,50^. Although the JMS contains high contents of minerals such as Na, Mg, Ca, K, Zn and strontium, the mechanism regarding how minerals absorbed during the bathing affects knee OA, is still uncertain, and further studies are needed.

To better understand bathing effects in the JMS on the pathophysiological process of knee OA, surgically induced rat model that reflects a post-traumatic early OA was employed. While the model has an advantage presenting reproducible disease progression, it can’t be entirely representative of human knee OA including the naturally degenerative changes. In addition, there has been inconsistent efficacy between animal and human studies, probably because of the differences in the anatomy, histology and physiology^51^. However, limitations in the clinical studies showing variations in the disease onset and the progression, necessitate a preclinical study on the early OA development for the fundamental cure. Even though just one animal model is not sufficient to study the effects of JMS bathing for all of knee OA, this may subsequently contribute to the development of treatment regimens. The current finding suggests beneficial effects of the JMS bathing mainly by inhibiting inflammation as a possible target. Given the global burden of OA disease requiring long-term management with various treatment options, this provides useful information supporting that JMS bathing can be a potent alternative therapy for improving quality of life in patients with knee OA.

## Methods

### JMS preparation

JMS was provided by the Korea Institute of Ocean Science and Technology (Ansan, Korea). It contained various minerals, such as Na (10,510 mg/L), Mg (1,295 mg/L), Ca (399 mg/L), K (400 mg/L), brom (63.4 mg/L), strontium (7.70 mg/L), Zn (0.019 mg/L), iron (0.015 mg/L), Mn (0.008 mg/L), V (0.015 mg/L), Se (0.013 mg/L), and germanium (0.002 mg/L). Its salinity was 33‰ similar to that of other seawaters.

### Animals and OA induction

Animal experiments were carried out according to the national regulations of the usage and welfare of laboratory animals and approved by the Institutional Animal Care and Use Committee of Daegu Haany University (Gyeongsan, Korea; Approval No. DHU2017-052). Six-week old Sprague-Dawley male rats were purchased from Orient Bio Inc. (Seungnam, Korea). They were housed five per a polycarbonate cage in a temperature (20–25 °C) and humidity (45–55%) controlled room with a light/dark cycle of 12/12 h. Food and water was available *ad libitum*. After acclimatization for 7 days, OA was surgically induced in the left knee as described previously^52^. Briefly, after an exposure of the medial joint capsule, the anterior cruciate ligament (ACL) was transected, and the medial meniscus was partially removed. The sham group received the same surgery except for the ACL transection and the partial meniscectomy.

### Experimental design

Four weeks after the surgical induction, the sham (n = 8) and OA model (n = 40) were selected based on body weights. Knee thickness was then measured by an electronic digital caliper (CD-15CPS, Mytutoyo, Tokyo, Japan), and the OA model was divided into five groups (n = 8 per group) with no significant differences in knee thickness (13.87 ± 0.18 mm in OA model, ranged in 13.61~14.22 mm; 10.96 ± 0.15 mm in sham, ranged in 10.76~11.17 mm). The sham and one group of OA model received an injection with saline as a vehicle of diclofenac sodium plus bathing in DW referring hydrotherpy (sham and OA control, respectively). The other four groups of OA model were allocated as treatment groups; an injection with diclofenac sodium (Wako Pure Chemical Industries, Ltd., Osaka, Japan) at 2 mg/kg in saline plus DW bathing (D2 + DW), saline injection plus JMS bathing (Sal + JMS) or injection of diclofenac sodium at 2 mg/kg and 1 mg/kg plus JMS bathing (D2 + JMS and D1 + JMS, respectively). The saline and diclofenac sodium were injected subcutaneously in a volume of 5 ml/kg, and the dosage was based on a previous study^52^. Thirty min after the administration, the rats underwent the bathing for 20 min in polycarbonate cages (280 × 420 × 180 mm) containing pre-warmed DW or JMS (4 L) at a depth of 5 cm under monitor to prevent accidental drinking of JMS. Rats received the treatment once a day for 8 weeks, and then euthanized using CO_2_ gas. The body weighs and knee thickness were measured every week by a technician blinded to the groups. Three days before euthanasia, the rats were intraperitoneally administered with BrdU (Sigma-Aldrich, St. Louise, MO, USA) at 50 mg/kg in a volume of 2 ml/kg^52^.

### Measurement of functional knee extension

The induced left hindlimb from the coxofemoral to the ankle region was sampled after all treatments. Thickness of the joint capsule and the maximum articular extension angle were measured as described previously^53^. The lower angle meant more functional improvement of the joint extension. This was performed by a veterinarian blinded to the groups.

### Measurement of BMD and compressive strength

BMD was assessed using DEXA (InAlyzer, MEDIKORS Inc., Seungnam, Korea) as described previously^54^, and expressed as g/cm^2^. It was measured in the knee joint and both subchondral bones of the femur and tibia by drawing boxes surrounding the approximate regions using the image analyzing soft-ware. Focal compressive strength was assessed in a central region of the medial femoral and tibial condyle at a depth of 0.2 mm using a computerized testing machine with a digital force gauge (JSV-H1000 and HF-10, Japan Instrumentation System Co., Tokyo, Japan) as Newton (*N*).

### Measurement of PGE2, 5-LO and MMPs

A part of the joint samples was homogenized in RIPA solution using a bead beater (Taco^TM^Prep, GeneReach Biotechnology Corp., Taichung, Taiwan) and an ultrasonic cell disruptor (KS-750, Madell Technology Corp., Ontario, CA, USA). The homogenates were centrifuged at 1,200 × g at 4 °C, and the supernatants were used for levels of PGE_2_, 5-LO, and MMPs. The PGE_2_ was measured at 450 nm using a PGE_2_ assay kit (PKGE004B, Parameter^TM^, R&D Systems, Minneapolis, MN, USA; detection of 39 to 2,500 pg/ml and sensitivity of 41.4 pg/ml), 5-LO activity was measured at 490 nm using a lipoxygenase inhibitor screening assay kit (760700, Cayman Chemical, Ann Arbor, MI, USA; detection of 0.5 to 5 nmol and sensitivity of 0.88 nmol), and MMP-2 and MMP-9 were measured at 560 nm using each ELISA kit (MBS494797; detection of 30 to 1,000 pg/ml and sensitivity of <10 pg/ml and MBS722532; sensitivity of 1.0 ng/ml, respectively, MyBioSource, San Diego, CA, USA), according to manufacturer’s instruction.

### Reverse transcription polymerase chain reaction

RNA was extracted using a TRIzol reagent (Invitrogen, Carlsbad, CA, USA), and the concentration and quality were determined by the CFX96^TM^ Real-Time System (Bio-Rad, Hercules, CA, USA). Contaminating DNA was removed by recombinant DNase I (DNA-free, Ambion, Austin, TX, USA). The RNA was reverse-transcribed using the reagent High-Capacity cDNA Reverse Transcription Kit (Applied Biosystems, Foster City, CA, USA). Then, cDNA products were mixed with specific primers as follows: for *collagen2*, 5′-GAGTGGAAGAGCGGAGACTACTG-3′ and 5′-CTCCA TGTTGCAGAAGACTTTCA-3′, for *SOX9*, 5′-AGAGCGTTGCTCGGAACTGT-3′ and 5′-TCCTGGACCGAAAC TGGTAAA-3′ and for *aggrecan*, 5′-GATGTCCCCTGCAATTACCA-3′ and 5′-TCTGTGCAAGTGA TTCGAGG-3′, as forward and reverse oligonucleotides, respectively. They were amplified in a condition of 58 °C for 30 min, 94 °C for 2 min, 35 cycles of 94 °C for 15 s, 60 °C for 30 s, 68 °C for 1 min, and then 72 °C for 5 min, using the ABI Step One Plus Sequence Detection System (Applied Biosystems). The expressions were normalized by β-actin as an internal control gene, and the relatives were analyzed using the delta-delta Ct method^55^. The primer for β-actin was forward 5′-ATCGTGGGCCGCCCTAGGCA-3′ and reverse 5′-TGGCCTTAGGGTT CAGAGGGG-3′.

### Histopathological analysis

A joint sample was fixed in 10% formalin and decalcified in a mixture of 24.4% formic acid and 0.5 N sodium hydroxide for 5 days. The samples were paraffin-embedded and serial-sectioned at a thickness of 3 μm. The sections were stained with Safranin O and evaluated according to the modified Mankin scores for four subgroups (0–3 points, each); surface of the cartilage, hypocellularity, cloning of cells and stain intensity^52^. The higher the score, the more severe the level of OA (semi-quantitative scores; max = 12). In addition, thicknesses of the articular cartilages (μm/cartilage), the synovial membrane-lining epithelial thickness (μm/knee joint) and inflammatory cells (cells/mm2) were assessed using a computerized image analyzer (iSolution FL v. 9.1, IMT i-solution Inc., Vancouver, BC, Canada). The histopathologist was blinded to the groups.

### Immunohistochemistry

Another serial section was pretreated with trypsin (Sigma-Aldrich) and 2 N hydrochloric acid for an antigen retrieval. The endogenous peroxidase was removed by 0.3% hydrogen peroxide, and the non-specific binding protein was treated with normal horse serum for 1 h. Then, the sections were incubated with primary antibodies at 4 °C overnight. The antibodies for COX-2 (160126, Cayman; 1:200), PARP (9545, Cell Signaling Technology Inc., Danvers, MA, USA; 1:100), TNF-α (sc-52746, Santa Cruz Biotechnology, Santa Cruz, CA, USA; 1:200) and BrdU (ab1893, Abcam, Cambridge, UK; 1:100) were used. Next day, the sections were incubated with a biotinylated secondary horse anti-mouse/rabbit IgG antibody and Vectastain Elite ABC reagents (Vector Laboratories Inc., Burlingame, CA, USA) for 1 h each. The immunoreactivity was visualized by a peroxidase substrate kit (Vector Laboratories Inc.). All sections were incubated in a humidity chamber, and rinsed with 0.01 M phosphate-buffered saline three times between each step. Cells occupying immunoreactive regions over 20% were regarded as positive, and the number was assessed in 10 regions of interest in each section by a histopathologist blinded to the groups.

### Statistical analyses

Data are expressed as means ± standard deviation (SD). First, data were analyzed by the Levene test for homogeneity of variance. If it is not significant, the values were examined by one way-ANOVA, followed by the least significant differences (LSD) post-hoc test. Otherwise, Kruskal-Wallis H test was conducted for non-parametric comparisons, followed by the Mann-Whitney U (MW) post-hoc with Bonferroni correction method. The kinetic data for the body weight and knee thickness were analyzed by two way-ANOVA with main factors of the groups and the time-point measured, and the time-point was treated as a repeated measurement. The multiple comparisons were focused on the effects of treatment groups including JMS bathing. A *p*-value <0.05 was considered statistically significant.

## Supplementary information

Supplementary figure S1.

## References

[1] Pereira D (2011). The effect of osteoarthritis definition on prevalence and incidence estimates: a systematic review. Osteoarthr. Cartil..

[2] Vos T (2012). Years lived with disability (YLDs) for 1160 sequelae of 289 diseases and injuries 1990-2010: a systematic analysis for the Global Burden of Disease Study 2010. Lancet.

[3] Man GS, Mologhianu G (2014). Osteoarthritis pathogenesis - a complex process that involves the entire joint. J. Med. Life.

[4] Nam J (2011). Sequential alterations in catabolic and anabolic gene expression parallel pathological changes during progression of monoiodoacetate-induced arthritis. PLoS One.

[5] Scanzello CR, Goldring SR (2012). The role of synovitis in osteoarthritis pathogenesis. Bone.

[6] Robinson WH (2016). Low-grade inflammation as a key mediator of the pathogenesis of osteoarthritis. Nat. Rev. Rheumatol..

[7] Hochberg MC (2012). American College of Rheumatology 2012 recommendations for the use of nonpharmacologic and pharmacologic therapies in osteoarthritis of the hand, hip, and knee. Arthritis Care Res..

[8] McAlindon TE (2014). OARSI guidelines for the non-surgical management of knee osteoarthritis. Osteoarthr. Cartil..

[9] Coxib (2013). Vascular and upper gastrointestinal effects of non-steroidal anti-inflammatory drugs: meta-analyses of individual participant data from randomised trials. Lancet.

[10] Roberts E (2016). Paracetamol: not as safe as we thought? A systematic literature review of observational studies. Ann. Rheum. Dis..

[11] Ungprasert P, Cheungpasitporn W, Crowson CS, Matteson EL (2015). Individual non-steroidal anti-inflammatory drugs and risk of acute kidney injury: A systematic review and meta-analysis of observational studies. Eur. J. Intern. Med..

[12] Cadet C, Maheu E, French AG (2014). Coxibs and traditional NSAIDs for pain relief. Lancet.

[13] Oo WM, Yu SP, Daniel MS, Hunter DJ (2018). Disease-modifying drugs in osteoarthritis: current understanding and future therapeutics. Expert. Opin. Emerg. Drugs.

[14] Bruyere O (2019). An updated algorithm recommendation for the management of knee osteoarthritis from the European Society for Clinical and Economic Aspects of Osteoporosis, Osteoarthritis and Musculoskeletal Diseases (ESCEO). Semin. Arthritis Rheum..

[15] Fioravanti A, Karagülle M, Bender T, Karagülle MZ (2017). Balneotherapy in osteoarthritis: facts, fiction and gaps in knowledge. Eur. J. Integr. Med..

[16] Ciani O (2017). Mud-Bath Therapy in Addition to Usual Care in Bilateral Knee Osteoarthritis: An Economic Evaluation Alongside a Randomized Controlled Trial. Arthritis Care Res..

[17] Vargas Negrin F, Medina Abellan MD, Hermosa Hernan JC, de Felipe Medina R (2014). Treatment of patients with osteoarthritis. Aten. Primaria.

[18] Fraioli A (2018). Efficacy of Spa Therapy, Mud-Pack Therapy, Balneotherapy, and Mud-Bath Therapy in the Management of Knee Osteoarthritis. A Systematic Review. Biomed. Res. Int..

[19] Forestier R (2010). Spa therapy in the treatment of knee osteoarthritis: a large randomised multicentre trial. Ann. Rheum. Dis..

[20] Antonelli M, Donelli D, Fioravanti A (2018). Effects of balneotherapy and spa therapy on quality of life of patients with knee osteoarthritis: a systematic review and meta-analysis. Rheumatol. Int..

[21] Matsumoto H (2017). The effect of balneotherapy on pain relief, stiffness, and physical function in patients with osteoarthritis of the knee: a meta-analysis. Clin. Rheumatol..

[22] Fioravanti A (2015). One-year follow-up of mud-bath therapy in patients with bilateral knee osteoarthritis: a randomized, single-blind controlled trial. Int. J. Biometeorol..

[23] Tefner IK (2013). The effect of Neydharting mud-pack therapy on knee osteoarthritis: a randomized, controlled, double-blind follow-up pilot study. Rheumatol. Int..

[24] Sukenik S, Flusser D, Abu-Shakra M (1999). The role of spa therapy in various rheumatic diseases. Rheum. Dis. Clin. North. Am..

[25] Flusser D, Abu-Shakra M, Friger M, Codish S, Sukenik S (2002). Therapy with mud compresses for knee osteoarthritis: comparison of natural mud preparations with mineral-depleted mud. J. Clin. Rheumatol..

[26] Fioravanti A (2015). Circulating levels of adiponectin, resistin, and visfatin after mud-bath therapy in patients with bilateral knee osteoarthritis. Int. J. Biometeorol..

[27] Benedetti S (2010). Biomarkers of oxidation, inflammation and cartilage degradation in osteoarthritis patients undergoing sulfur-based spa therapies. Clin. Biochem..

[28] Dönmez, A. *et al*. Effect of Mild Heat Stress on Heat Shock Protein 70 in a Balneotherapy Model. (2017).

[29] Kim B-Y, Lee Y-K, Park D-B (2012). Metabolic activity of desalted ground seawater of Jeju in rat muscle and human liver cells. Fish. Aquat. Sci..

[30] Lee, H. *et al*. Beneficial Effects of Desalinated Magma Seawater in Ameliorating Thioacetamide-induced Chronic Hepatotoxicity. *Biotechnology and Bioprocess Engineering*, 10.1007/s12257-018-0371-9 (2019).

[31] Kim, C. G. *et al*. Bathing effects of various seawaters on allergic (atopic) dermatitis-like skin lesions induced by 2, 4-dinitrochlorobenzene in hairless mice. *Evidence-Based Complementary and Alternative Medicine* **2015** (2015). 10.1155/2015/179185 PMC4488017 26221169

[32] Kim CG (2017). Bathing effects of east saline groundwater concentrates on allergic (atopic) dermatitis-like skin lesions induced by 2,4-dinitrochlorobenzene in hairless mice. Exp. Ther. Med..

[33] Halevy S (2001). The role of trace elements in psoriatic patients undergoing balneotherapy with Dead Sea bath salt. Isr. Med. Assoc. J..

[34] Beer A-M, Junginger H, Lukanov J, Sagorchev P (2003). Evaluation of the permeation of peat substances through human skin *in vitro*. Int. J. Pharmaceutics.

[35] Edd, S. N., Omoumi, P., Andriacchi, T. P., Jolles, B. M. & Favre, J. Modeling knee osteoarthritis pathophysiology using an integrated joint system (IJS): A systematic review of relationships among cartilage thickness, gait mechanics, and subchondral bone mineral density. *Osteoarthritis Cartilage*, 10.1016/j.joca.2018.06.017 (2018). 10.1016/j.joca.2018.06.017 30056214

[36] Sokolove J, Lepus CM (2013). Role of inflammation in the pathogenesis of osteoarthritis: latest findings and interpretations. Ther. Adv. Musculoskelet. Dis..

[37] Furumatsu T, Tsuda M, Taniguchi N, Tajima Y, Asahara H (2005). Smad3 induces chondrogenesis through the activation of SOX9 via CREB-binding protein/p300 recruitment. J. Biol. Chem..

[38] Zeng GQ, Chen AB, Li W, Song JH, Gao CY (2015). High MMP-1, MMP-2, and MMP-9 protein levels in osteoarthritis. Genet. Mol. Res..

[39] Pearle AD, Warren RF, Rodeo SA (2005). Basic science of articular cartilage and osteoarthritis. Clin. Sports Med..

[40] Tamura T, Ohmori K (2001). Rhein, an active metabolite of diacerein, suppresses the interleukin-1alpha-induced proteoglycan degradation in cultured rabbit articular chondrocytes. Jpn. J. Pharmacol..

[41] Balint GP (2007). The effect of the thermal mineral water of Nagybaracska on patients with knee joint osteoarthritis–a double blind study. Clin. Rheumatol..

[42] Kovacs I, Bender T (2002). The therapeutic effects of Cserkeszolo thermal water in osteoarthritis of the knee: a double blind, controlled, follow-up study. Rheumatol. Int..

[43] Galvez, I., Torres-Piles, S. & Ortega-Rincon, E. Balneotherapy, Immune System, and Stress Response: A Hormetic Strategy? *Int J Mol Sci* **19**, 10.3390/ijms19061687 (2018). 10.3390/ijms19061687 PMC6032246 29882782

[44] Frestedt JL, Walsh M, Kuskowski MA, Zenk JL (2008). A natural mineral supplement provides relief from knee osteoarthritis symptoms: a randomized controlled pilot trial. Nutr. J..

[45] Kurz B, Jost B, Schunke M (2002). Dietary vitamins and selenium diminish the development of mechanically induced osteoarthritis and increase the expression of antioxidative enzymes in the knee joint of STR/1N mice. Osteoarthr. Cartil..

[46] El Karib AO (2016). Insulin and vanadium protect against osteoarthritis development secondary to diabetes mellitus in rats. Arch. Physiol. Biochem..

[47] Nielsen FH (2018). Magnesium deficiency and increased inflammation: current perspectives. J. Inflamm. Res..

[48] Zeng C (2015). Association between Dietary Magnesium Intake and Radiographic Knee Osteoarthritis. PLoS One.

[49] Liu HY (2013). Potential Osteoporosis Recovery by Deep Sea Water through Bone Regeneration in SAMP8 Mice. Evid. Based Complement. Altern. Med..

[50] Maehira F, Iinuma Y, Eguchi Y, Miyagi I, Teruya S (2008). Effects of soluble silicon compound and deep-sea water on biochemical and mechanical properties of bone and the related gene expression in mice. J. Bone Min. Metab..

[51] Kuyinu EL, Narayanan G, Nair LS, Laurencin CT (2016). Animal models of osteoarthritis: classification, update, and measurement of outcomes. J. Orthop. Surg. Res..

[52] Choi JS (2015). Effect of Polycalcium, a mixture of Polycan and calcium lactate-gluconate in a 1:9 weight ratio, on rats with surgery-induced osteoarthritis. Exp. Ther. Med..

[53] Rezende MU (2006). Diacerhein versus glucosamine in a rat model of osteoarthritis. Clinics.

[54] Kang SJ (2017). Anti-climacterium effects of pomegranate concentrated solutions in ovariectomized ddY mice. Exp. Ther. Med..

[55] Chen CG, Thuillier D, Chin EN, Alliston T (2012). Chondrocyte-intrinsic Smad3 represses Runx2-inducible matrix metalloproteinase 13 expression to maintain articular cartilage and prevent osteoarthritis. Arthritis Rheum..

