# Supplementary material for: Effects of Balneotherapy in Jeju Magma-Seawater on Knee Osteoarthritis Model
Source: Sci Rep. 2020 Apr 20;10:6620. doi: 10.1038/s41598-020-62867-2 (PMC7171195; doi:10.1038/s41598-020-62867-2)
Supplement: Supplementary file 1 — Supplementary figure S1. [file 41598_2020_62867_MOESM1_ESM.pdf]

RESEARCH ARTICLE

Effects of Balneotherapy in Jeju Magma-Seawater on Knee Osteoarthritis Model

Short title: Bathing effects in JMS on knee OA and the synergic effects with diclofenac sodium

Choong-Gon Kim[1¶] (kimcg@kiost.ac.kr), Dae-Geon Lee[2¶] (ghost71715@naver.com), Jina Oh[1] (jnoh@kiost.ac.kr), Youn-Ho Lee[1] (ylee@kiost.ac.kr), Young Joon Lee[3] (gksxntk@dhu.ac.kr), Phil Hyun Song[4] (sph04@hanmail.net), Chang-Hyun Song[2*] (dvmsong@hotmail.com), and Sae-Kwang Ku[2*] (gucci200@hanmail.net)

[1] Marine Ecosystem and Biological Research Center, Korea Institute of Ocean Science and Technology, Busan, Republic of Korea

[2] Department of Anatomy and Histology, College of Korean Medicine, Daegu Haany University, Gyeongsan, Gyeongsangbuk-do, Republic of Korea

[3] Department of Preventive Medicine, College of Korean Medicine, Daegu Haany University, Gyeongsan, Gyeongsangbuk-do, Republic of Korea

[4] Department of Urology, College of Medicine, Yeungnam University, Daegu, Republic of Korea

[*] Corresponding authors

E-mail: dvmsong@hotmail.com (C.-H.S.) and gucci200@hanmail.net (S.-K.K.)

[¶] These authors contributed equally to this work.

Figure S1

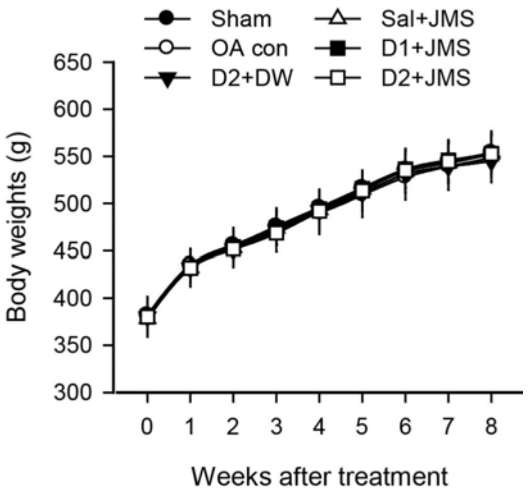

Supplementary figure S1. Body weight changes. Osteoarthritis (OA) rat model received a subcutaneous injection of saline (Sal) or diclofenac sodium at 1 mg/kg (D1) or 2 mg/kg (D2), followed by bathing in thermal distilled water (DW) or Jeju magma-seawater (JMS) for 8 weeks. The group was designated according to a treatment of the injection plus bathing. The OA control (OA con) and the corresponding sham received Sal plus DW bathing. Body weight was measured every week, and values were expressed as means ± standard deviation (eight rats/group).
